# FPGA Implementation of the Chirp-Scaling Algorithm for Real-Time Synthetic Aperture Radar Imaging

**DOI:** 10.3390/s23020959

**Published:** 2023-01-14

**Authors:** Jaehyeon Lee, Dongmin Jeong, Seongwook Lee, Myeongjin Lee, Wookyung Lee, Yunho Jung

**Affiliations:** 1 School of Electronics and Information Engineering, Korea Aerospace University, Goyang-si 10540, Republic of Korea; 2 Department of Smart Air Mobility, Korea Aerospace University, Goyang-si 10540, Republic of Korea

**Keywords:** synthetic aperture radar (SAR), chirp-scaling algorithm (CSA), real-time processing, systolic array processor, field programmable gate array (FPGA)

## Abstract

Synthetic aperture radar (SAR), which can generate images of regions or objects, is an important research area of radar. The chirp scaling algorithm (CSA) is a representative SAR imaging algorithm. The CSA has a simple structure comprising phase compensation and fast Fourier transform (FFT) operations by replacing interpolation for range cell migration correction (RCMC) with phase compensation. However, real-time processing still requires many computations and a long execution time. Therefore, it is necessary to develop a hardware accelerator to improve the speed of algorithm processing. In addition, the demand for a small SAR system that can be mounted on a small aircraft or drone and that satisfies the constraints of area and power consumption is increasing. In this study, we proposed a CSA-based SAR processor that supports FFT and phase compensation operations and presents field-programmable gate array (FPGA)-based implementation results. We also proposed a modified CSA flow that simplifies the traditional CSA flow by changing the order in which the transpose operation occurs. Therefore, the proposed CSA-based SAR processor was designed to be suitable for modified CSA flow. We designed the multiplier for FFT to be shared for phase compensation, thereby achieving area efficiency and simplifying the data flow. The proposed CSA-based SAR processor was implemented on a Xilinx UltraScale+ MPSoC FPGA device and designed using Verilog-HDL. After comparing the execution times of the proposed SAR processor and the ARM cortex-A53 microprocessor, we observed a 136.2-fold increase in speed for the 4096 × 4096-pixel image.

## 1. Introduction

**Figure 1:**
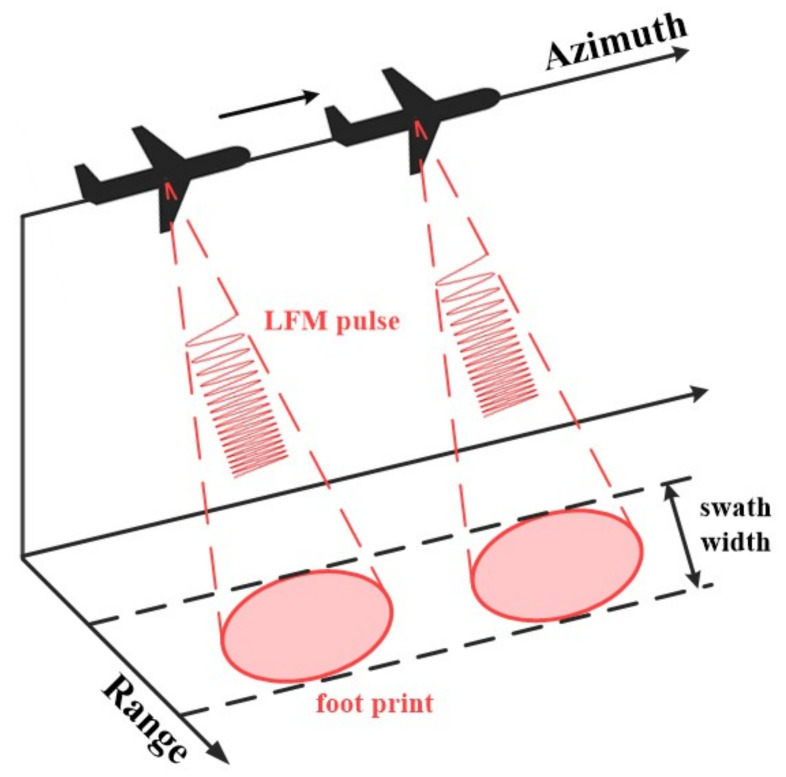
Illustration of the working principle of SAR.

The synthetic aperture radar (SAR) is an active sensor system that can acquire high-resolution radar images, regardless of day or night, flight altitude, and weather, using a microwave band [1,2,3]. Figure 1 shows the working principle of the SAR. In the SAR system, a small antenna is mounted on a platform that moves along a flight path. The direction of flight is also called the azimuth direction, and the range direction is perpendicular to the azimuth direction. The direction of the antenna is a range direction, and it moves by illuminating an area called a swath. Two-dimensional data on the azimuth and the range are collected by transmitting and receiving pulses. The central idea of the SAR is based upon matching filtering for both the azimuth and distance directions, which results in high-resolution radar images. In addition, the SAR has the advantage of remote sensing, so it plays a vital role in various fields, such as disaster emergency response, environmental protection, and military applications [3,4,5,6]. Because the traditional SAR system requires considerable computing resources and high power consumption, it has been mounted on large platforms such as aircraft and satellites. However, recent advances in digital signal processing and complementary metal oxide semiconductor (CMOS) technologies have made it possible to develop small and lightweight SAR systems. Accordingly, research on SAR systems with low power consumption and real-time processing is increasing [7,8,9].

Operations for SAR imaging mainly include the fast Fourier transform (FFT), inverse fast Fourier transform (IFFT), phase compensation, interpolation, etc., and the computational complexity of these operations is very high. Therefore, real-time SAR imaging necessitates accelerating these operations on various computing platforms, such as the central processing unit (CPU), the graphic processing unit (GPU), the field-programmable gate array (FPGA), and application-specific integrated circuits (ASICs) [9,10,11,12,13,14,15,16,17,18]. CPU and GPU provide high flexibility for software through various instructions and show high performance in single and parallel processing, respectively. However, high power consumption is still a severe challenge. On the other hand, FPGA has latency, throughput, and power consumption advantages compared with CPU or GPU. In addition, it has gained attention as a computing platform that can be used in various fields owing to its high flexibility [19,20].

Several studies have been conducted on the implementation of SAR systems using FPGAs. In 2004, Le et al. proposed an FPGA-based hardware architecture for a spaceborne system to process the range-Doppler and space–time adaptive processing (STAP) algorithms [9]. Greco et al. proposed an HW/SW interface framework to use FPGA resources efficiently through an abstraction layer and verified it in SAR applications and confirmed its performance [10]. Pfitzner et al. proposed an FPGA-based hardware architecture for airborne, real-time SAR imaging with integrated first-order motion compensation (MoCom) [11]. Lou et al. proposed a UAVSAR onboard processor for real-time and autonomous operations. They demonstrated the use of UAVSAR data to determine the flood extent, forest fire extent, lava flow, and landslide [12]. Choi et al. proposed a range-Doppler algorithm (RDA)-based SAR processor for real-time SAR imaging. In the case of RDA, interpolation is performed for range cell migration correction (RCMC). Therefore, all operations of RDA are accelerated by implementing an RCMC unit in addition to the FFT unit. However, the FFT unit adopts a pipelined structure, so there is room for speed improvement [13].

The most commonly used SAR Imaging algorithms include range-Doppler, chirp scaling, omega-K, polar format, and back projection. The RDA performs efficient imaging through block processing in the range and azimuth frequency domains; however, the complexity of interpolation for RCMC is very high. Therefore, the chirp-scaling algorithm (CSA) was developed by replacing the interpolation of RDA with phase compensation. CSA has a simple algorithm structure comprising FFT and phase compensation operations. In addition, CSA has an advantage of real-time imaging because it has the smallest computational load compared with the RDA and omega-K algorithms [21].

Several studies have been conducted to implement CSA on various platforms. Zhang et al. proposed a collaborative SAR imaging method that performs efficient task partitioning and scheduling. The entire image can be generated using deep collaborative multiple CPU–GPU computing. It acquired a 32,728 × 32,728-pixel image in 2.8 s [14]. Tang et al. proposed a simulator for spaceborne SAR onboard imaging on mobile GPUs. It acquired a 4096 × 4096-pixel image in 14.97 s [15]. Wang et al. proposed a heterogeneous processor consisting of fixed-point PE units and floating-point PE units. It acquired a 32,768 × 32,768-pixel image in 32.9 s at a speed of 200 MHz [16]. Li et al. proposed a method that employs single-instruction, multiple-data (SIMD) instructions and open multiprocessing (OpenMP) technology on multicore SIMD CPU to realize parallel optimization on CSA [17]. Di et al. proposed a schedulable and scalable multicore parallel architecture based on FPGA and mapped the fundamental CSA to the system. It acquired a 1024 × 4096-pixel image in 12 s [18].

Among the CSA operations, FFT/IFFT operations account for the highest proportion. Therefore, it is necessary to implement an FFT/IFFT processor for real-time imaging. The hardware structure of the FFT processor is divided into the butterfly, pipeline, and systolic array structures [22,23,24]. Butterfly and pipeline structures can be implemented with fewer hardware resources but are unsuitable for high-speed operations. Therefore, a systolic array-based FFT processor is suitable for real-time imaging [25,26]. Among the various systolic array structures, the base-4 systolic array structure is arithmetically efficient and has a good trade-off between area and speed [27,28]. Therefore, We adopted the base-4 systolic array structure.

In this paper, we propose a CSA-based SAR processor and present the results of accelerating the modified CSA flow, in which the order of transpose operation is changed in the traditional CSA flow. The proposed CSA-based SAR processor was implemented based on a base-4 systolic array architecture and can only perform FFT or FFT and phase compensation operations simultaneously. Twiddle factor multiplication and phase compensation were designed to share the same multiplier owing to their commonality of element-by-element multiplication, which made it possible to simplify the data flow and achieve area efficiency.

The remainder of this paper is organized as follows: Section 2 reviews the CSA and base-b FFT algorithm. Section 3 describes the modified CSA algorithm and the hardware architecture of the proposed CSA-based SAR processor. Section 4 presents the proposed processor’s implementation and the accelerated CSA results and compares the speed performance with previous studies. Finally, Section 5 concludes the paper.

## 2. Background

### 2.1. Chirp Scaling Algorithm

The CSA is one of the most popular algorithms for SAR imaging. It is instrumental because it can support strip-map, scan SAR, spotlight, sliding spotlight, tops, and mosaic modes, along with other pre- and post-processing steps [29,30]. The CSA operation has a simple algorithm structure comprising only FFT and phase compensation operations. Because signal processing is possible in the two-dimensional frequency domain, it is possible to solve the problem of secondary range compression (SRC), which depends on azimuth frequency. The computational complexity was reduced by replacing the interpolation operation for RCMC with phase compensation, which was performed in two steps: differential RCMC and bulk RCMC.

**Figure 2:**
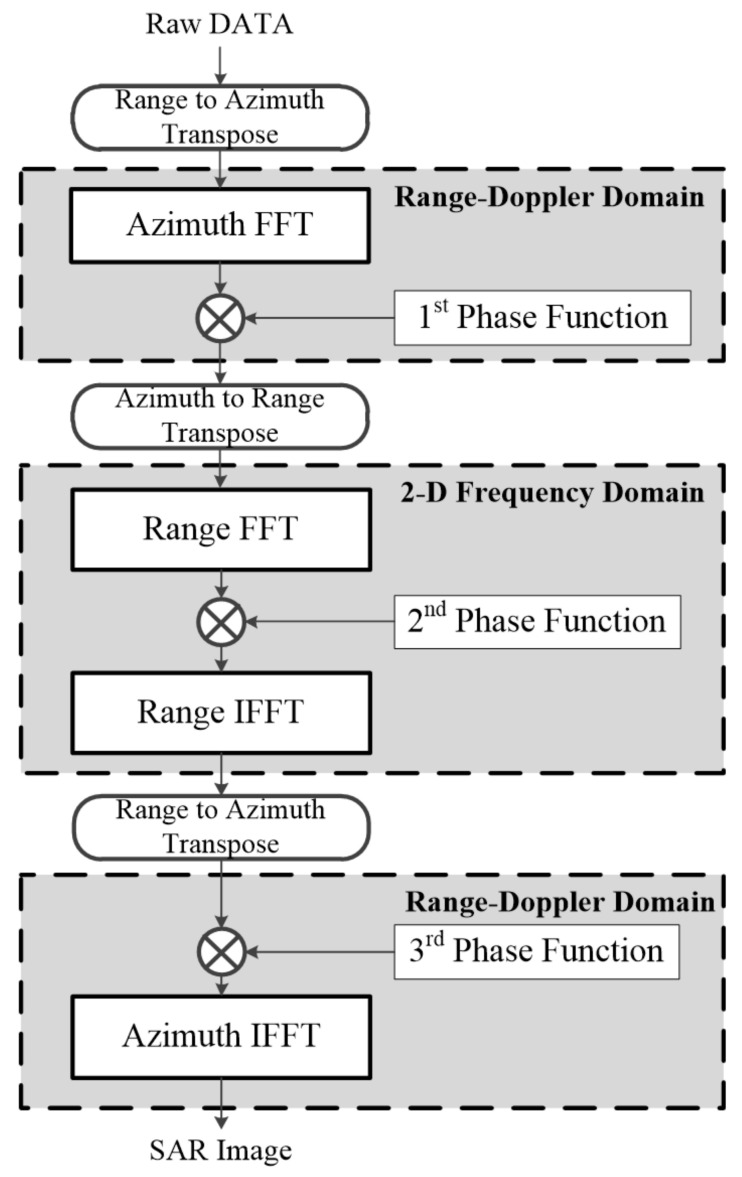
Traditional CSA flow.

The traditional CSA flow is shown in Figure 2. SAR images can be obtained using four times FFT/IFFT and three times phase compensation operations. Differential RCMC to achieve chirp scaling is performed with the first-phase function, and bulk RCMC and range compression are performed using the second-phase function. Finally, the SAR image can be obtained through the third-phase function by performing azimuth compression and compensating for the residual phase.

The transmission signal of a pulse-Doppler radar is assumed to be a linear frequency modulation (FM) chirp signal. The signal converted into the range-Doppler domain through the azimuth FFT is shown in Equation (1). Thus, all the targets in the same range of the closest approach collapse into one trajectory in the azimuth frequency domain [31].

(1) $$\begin{array}{cc} \; & s_{rd}\left(\tau ,f_{\eta }\right)=Aw_{r}\left[\tau -\frac{2R_{0}}{cD\left(f_{\eta },V_{r}\right)}\right]W_{a}\left(f_{\eta }-f_{\eta_{c}}\right)\\ & \;\;\;\times \exp\left[-j\frac{4\pi f_{0}R_{0}D\left(f_{\eta },V_{r}\right)}{c}\right]\times \exp\left[j\pi K_{m}{\left(\tau -\frac{2R_{0}}{cD\left(f_{\eta },V_{r}\right)}\right)}^{2}\right] \end{array}$$

where τ is the range time, $f_{\eta }$ is the azimuth frequency, *A* is the complex constant, *c* is the speed of light, $D\left(.\right)$ is the migration factor in the range-Doppler domain, $V_{r}$ is the effective radar velocity, $R_{0}$ is the slant range of closest approach, $f_{\eta_{c}}$ is the azimuth FM rate of the point target signal, $f_{0}$ is the carrier frequency, and $K_{m}$ is the range FM of the point target signal in the range-Doppler domain. To adjust the range movement of the trajectory through the differential RCMC, phase compensation is performed using the first-phase function expressed by Equation (2), and the result can be expressed as Equation (3).

(2) $$s_{sc}\left(\tau^{\prime },f_{\eta }\right)=\exp\left[j\pi K_{m}\left(\frac{D\left(f_{\eta_{ref}},V_{r_{ref}}\right)}{D\left(f_{\eta },V_{r_{ref}}\right)}\right){\left(\tau^{\prime }\right)}^{2}\right]$$

where $f_{\eta_{ref}}$ is the reference azimuth frequency, and $V_{r_{ref}}$ is the effective radar velocity in the reference range.

(3) $$s_{1}\left(\tau ,f_{\eta }\right)=s_{sc}\left(\tau^{\prime },f_{\eta }\right)S_{rd}\left(\tau ,f_{\eta }\right)$$

Equation (3) is transformed into a two-dimensional frequency domain with a range FFT, resulting in the signal given by Equation (4). There are five exponential terms, and compensation for these terms is performed through subsequent processing processes.

(4) $$\begin{array}{cc} \;s_{2}\left(f_{\tau },f_{\eta }\right)= & A_{1}W_{r}\left(f_{\tau }\right)W_{a}\left(f_{\eta }-f_{\eta_{c}}\right)\times \exp\left[-j\frac{4\pi f_{0}R_{0}D\left(f_{\eta },V_{r}\right)}{c}\right]\\ \; & \times \exp\left[-j\frac{\pi D\left(f_{\eta },V_{r}\right)}{K_{m}D\left(f_{\eta_{ref}},V_{r}\right)}f_{\tau }^{2}\right]\times \exp\left[-j\frac{4\pi R_{0}}{cD\left(f_{\eta_{ref}},V_{r_{ref}}\right)}f_{\tau }\right]\\ \; & \times \exp\left[-j\frac{4\pi }{c}\left(\frac{1}{D\left(f_{\eta },V_{r_{ref}}\right)}-\frac{1}{D\left(f_{\eta_{ref}},V_{V_{ref}}\right)}\right)R_{ref}f_{\tau }\right]\\ \; & \times \exp\left[j\frac{4\pi K_{m}}{c^{2}}\left(1-\frac{D\left(f_{\eta },V_{r_{ref}}\right)}{D\left(f_{\eta_{ref}},V_{r_{ref}}\right)}\right)\times {\left(\frac{R_{0}}{D\left(f_{\eta },V_{r}\right)}-\frac{R_{ref}}{D\left(f_{\eta },V_{r}\right)}\right)}^{2}\right] \end{array}$$

where $A_{1}$ is the complex constant, and $f_{\tau }$ is the range frequency. The second exponential term represents the range modulation after the scaling and includes the range–azimuth coupling corrected by the SRC. The fourth exponential term represents bulk range cell migration. The second-phase function performs range compression, SRC, and bulk RCMC by compensating for these two terms. The result is given by Equation (5).

(5) $$\begin{array}{cc} \; & s_{3}\left(f_{\tau },f_{\eta }\right)=A_{1}W_{\tau }\left(f_{\tau }\right)W_{a}\left(f_{\eta }-f_{\eta_{c}}\right)\\ \; & \;\;\;\;\times exp\left[-j\frac{4\pi f_{0}R_{0}D\left(f_{\eta },V_{r}\right)}{c}\right]\times exp\left[-j\frac{4\pi R_{0}}{cD\left(f_{\eta_{ref}},{\;V}_{r_{ref}}\right)}f_{\tau }\right]\\ \; & \;\;\;\;\times exp\left[j\frac{4\pi K_{m}}{c^{2}}\left(1-\frac{D\left(f_{\eta },\;V_{r_{ref}}\right)}{D\left(f_{\eta_{ref}},V_{r_{ref}}\right)}\right)\right.\left.\times {\left(\frac{R_{0}}{D\left(f_{\eta },V_{r}\right)}-\frac{R_{ref}}{D\left(f_{\eta },V_{r}\right)}\right)}^{2}\right] \end{array}$$

Next, range IFFT is performed to transform the signal into the range-Doppler domain, and the result is given by Equation (6).

(6) $$\begin{array}{cc} \; & s_{4}\left(\tau ,f_{\eta }\right)=A_{2}p_{r}\left(\tau -\frac{2R_{0}}{cD\left(f_{\eta_{ref}},V_{r_{ref}}\right)}\right)W_{a}\left(f_{\eta },f_{\eta_{c}}\right)\times \exp\left[-j\frac{4\pi R_{0}f_{0}D\left(f_{\eta },V_{r}\right)}{c}\right]\\ & \;\;\;\times \exp\left[j\frac{4\pi K_{m}}{c^{2}}\left(1-\frac{D\left(f_{\eta },V_{r_{ref}}\right)}{D\left(f_{\eta_{ref}},V_{r_{ref}}\right)}\right)\times {\left(\frac{R_{0}}{D\left(f_{\eta },V_{r}\right)}-\frac{R_{ref}}{D\left(f_{\eta },V_{r}\right)}\right)}^{2}\right] \end{array}$$

where $A_{2}$ is the complex constant, and $P_{r}\left(\tau \right)$ is the range envelope. By multiplying Equation (6) by the third-phase function, the first exponential term representing the azimuth modulation and the second exponential term representing the residual phases can be compensated. Finally, azimuth IFFT is performed to transform the signal into the time domain. The signal of the point target is given by Equation (7).

(7) $$s_{5}\left(\tau ,\eta \right)=A_{4}p_{r}\left[\tau -\frac{2R_{0}}{cD\left(f_{\eta_{ref}},V_{r_{ref}}\right)}\right]P_{a}\left(\eta -\eta_{c}\right)\times exp\left[j\theta \left(\tau ,\eta \right)\right]$$

where $A_{4}$ is the complex constant, $P_{a}\left(\eta \right)$ is the IFFT of the window $W_{a}\left(f_{\eta }\right)$, and $\theta \left(\tau ,\eta \right)$ is the target phase.

### 2.2. Base-*b* FFT Algorithm

We adopted a base-*b* FFT algorithm based on two levels of transform factorization to compute the discrete Fourier transform (DFT) [28]. A DFT of length *N* is given by Equation (8).

(8) $$Z\left(k\right)=\sum_{n=0}^{N-1}W_{N}^{nk}X\left(n\right),\;n,k=0,1,\cdots ,N-1$$

where $X\left(n\right)$ are the time-domain input values, $Z\left(k\right)$ are the frequency-domain outputs, and $W_{N}^{nk}$ is the twiddle factor, $e^{-j\frac{2\pi }{N}nk}$. The matrix form of Equation (8) is given by Equation (9).

(9) Z=CX

where *C* is the coefficient matrix containing the twiddle factor.

If the one-dimensional input data of length *N* can be decomposed into rows and columns, $N=N_{1}N_{2}$, *n*, and *k* can be represented by Equation (10). By substituting Equation (10), Equation (8) can be expressed as Equation (11).

(10) $$\begin{array}{cc} \; & n=n_{1}+N_{1}n_{2},\left(0\leq n_{1}\leq N_{1}-1,0\leq n_{2}\leq N_{2}-1\right)\\ \; & k=k_{1}+N_{1}k_{2},\left(0\leq k_{1}\leq N_{1}-1,0\leq k_{2}\leq N_{2}-1\right) \end{array}$$

(11) $$Z\left(k_{1}+N_{1}k_{2}\right)=\sum_{n_{1}=0}^{N_{1}-1}\left(W_{N}^{n_{1}k_{1}}\sum_{n_{2}=0}^{N_{2}-1}W_{N_{2}}^{n_{2}k_{1}}W_{N_{2}}^{n_{2}k_{2}N_{1}}X\left(n_{1}+N_{1}n_{2}\right)\right)W_{N_{2}}^{n_{1}k_{2}}$$

Equation (11) can be simplified to Equation (12) by constraining $N_{1}/N_{2}$ as an integer value such that $W_{N_{2}}^{n_{2}k_{2}N_{1}}=e^{-j\frac{2\pi n_{2}k_{2}N_{1}}{N_{2}}}=1$.

(12) $$Z\left(k_{1}+N_{1}k_{2}\right)=\sum_{n_{1}=0}^{N_{1}-1}\left(W_{N}^{n_{1}k_{1}}\sum_{n_{2}=0}^{N_{2}-1}W_{N_{2}}^{n_{2}k_{1}}X\left(n_{1}+N_{1}n_{2}\right)\right)W_{N_{2}}^{n_{1}k_{2}}$$

For any particular value of n and k, the inner parenthesis value of Equation (12) can be evaluated in the dot product, as in Equation (13).

(13) $$Y\left(k_{1},n_{1}\right)=W_{N}^{n_{1}k_{1}}\left[W_{N_{2}}^{0}W_{N_{2}}^{k_{1}}W_{N_{2}}^{2k_{1}}\ldots W_{N_{2}}^{\left(N_{2}-1\right)k_{1}}\right]\times \left[\begin{array}{c} X\left(n_{1}\right)\\ X\left(n_{1}+N_{1}\right)\\ X\left(n_{1}+2N_{1}\right)\\ \vdots \\ X\left(n_{1}+\left(N_{2}-1\right)N_{1}\right) \end{array}\right]$$

With Equation (13), Equation (12) becomes Equation (14):

(14) $$Z\left(k_{1}+N_{1}k_{2}\right)=\sum_{n_{1}=0}^{N_{1}-1}Y\left(k_{1},n_{1}\right)W_{N_{2}}^{n_{1}k_{2}}$$

The matrix form of *Y* is given by Equation (15). $Y\left({k_{1},n}_{1}\right)$ values for all $n_{1}$, $k_{1}$ can be expressed as a matrix of size $N_{1}\times N_{1}$. $W_{M}$ is a matrix of size $N_{1}\times N_{1}$ and represents $W_{N}^{n_{1}k_{1}}$, and · means element-by-element multiplication. $C_{M1}$ is a matrix of size $N_{1}\times N_{2}$ and represents $W_{N_{2}}^{n_{2}k_{1}}$. Because *X* representing $X\left(n_{1}+N_{1}n_{2}\right)$ is a matrix of size $N_{2}\times N_{1}$, *Y* becomes a matrix of size $N_{1}\times N_{1}$.

(15) $$Y=W_{M}\cdot C_{M1}X$$

Similarly to *Y*, the *Z* can be calculated from the dot product, as shown in Equation (16). Consequently, the matrix form for calculating *Z* is given by Equation (17).

(16) $$Z\left(k_{1}+N_{1}k_{2}\right)=\left[\begin{array}{ccccc} W_{N_{2}}^{0} & W_{N_{2}}^{k_{2}} & W_{N_{2}}^{2k_{2}} & \ldots & W_{N_{2}}^{\left(N_{2}-1\right)k_{2}} \end{array}\right]\times \left[\begin{array}{c} Y\left(k_{1},0\right)\\ Y\left(k_{1},1\right)\\ Y\left(k_{1},2\right)\\ \vdots \\ Y\left(k_{1},\left(N_{1}-1\right)\right) \end{array}\right]$$

(17) $$Z=C_{M2}Y^{t}$$

where $C_{M2}$ is a coefficient matrix of size $N_{2}\times N_{1}$ and represents $W_{N_{2}}^{n_{1}k_{2}}$, which is equivalent to the transpose of $C_{M1}$. *Z* is a matrix of size $N_{2}\times N_{1}$ and represents the result of the DFT.

In the base-*b* FFT algorithm, *b* is the value of $N_{2}$, which can be used as a different value depending on the application. The base-*b* FFT algorithm is performed using two levels of transform factorization for one-dimensional data of length *N*. The first factorization is performed such that $N=N_{r}N_{c}$ using the traditional row/column approach to lower the computational complexity. The second factorization is performed to $N_{r}=N_{1r}N_{2}$ and $N_{c}=N_{1c}N_{2}$. FFT is performed through Equations (15) and (17) using the factorized result as an input.

FFT is performed in three steps. Column FFT is performed $N_{r}$ times in the row direction using column data with a length of $N_{c}$. Next, the result of column FFT is multiplied by $W_{N}$. Finally, row FFT is performed $N_{c}$ times in the column direction for row data with a length of $N_{r}$. In summary, after transforming the one-dimensional data into a two-dimensional matrix of size $N_{r}\times N_{c}$, column FFT, $W_{N}$ multiplication, and row FFT are performed to obtain the FFT results.

## 3. Proposed HW Architecture

**Figure 3:**
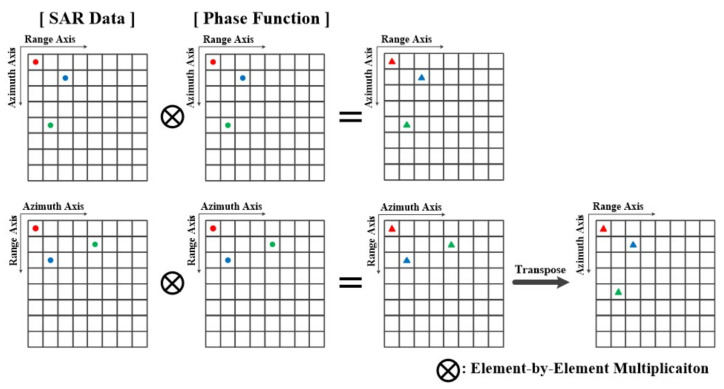
Phase compensation operation with transposed data.

The CSA includes an FFT operation, which is a vector operation, and a phase compensation operation, which is a scalar operation (element-by-element multiplication). Therefore, for phase compensation, the desired result can be obtained by matching the axes of the SAR data and the phase function. Figure 3 shows the phase compensation operation with transposed data. The first row shows the operation results on the range axis, and the second row shows the operation results on the azimuth axis. The transpose of the result in the second row is the same as that in the first row.

**Figure 4:**
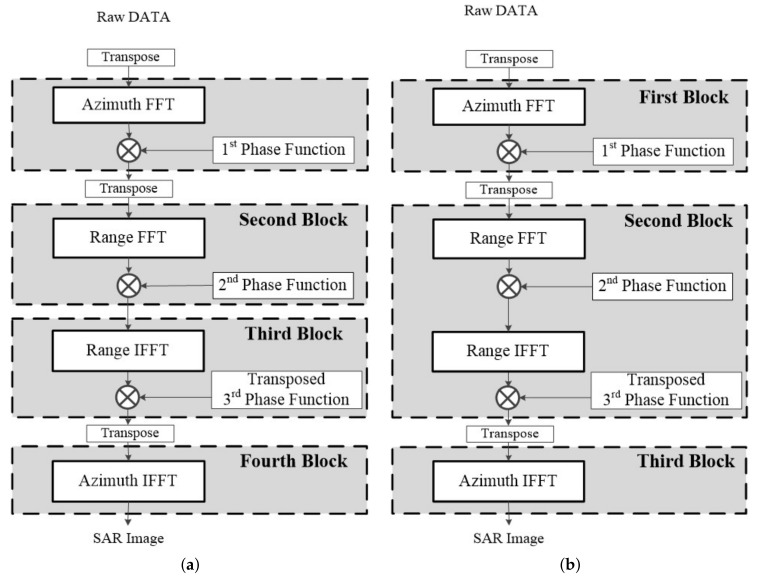
Modified CSA flows: (**a**) modified 1; (**b**) modified 2.

By performing transpose for the phase function, we changed the order in which data is transposed in the traditional CSA flow. Figure 4 shows the proposed modified CSA flow. We transposed the third-phase function and changed the transpose operation of the data from after range IFFT to after the third-phase compensation. The difference is that the third-phase compensation was performed on the range axis. FFT/IFFT and phase compensation operations were repeated three times as a new operation block, and then azimuth IFFT was performed to obtain SAR images. In the modified CSA flow 2, because the second and third blocks were both processed on the range axis, there was no need to store the data in the external memory to transpose the data. Accordingly, modified flow 2, which integrated the second and third blocks, was determined as the CSA processing flow.

**Figure 5:**
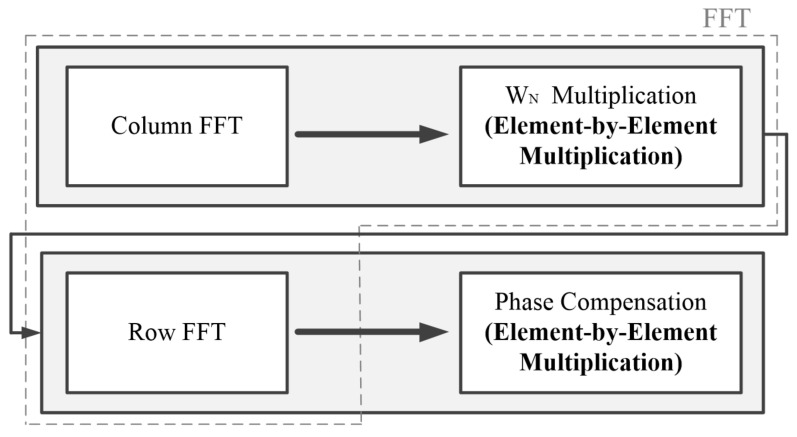
Procedure of FFT and phase compensation.

Figure 5 shows the FFT and phase compensation procedure, which is a repeated operation block in the modified CSA flow. The block operation proceeded in the order of column FFT, $W_{N}$ multiplication, row FFT, and phase compensation. Both the $W_{N}$ multiplication and phase compensation operations were element-by-element multiplications. Therefore, by repeating the row or column FFT and element-by-element multiplication twice, FFT and phase compensation could be performed. Thus, the operation block was accelerated by subdividing the FFT and the phase compensation operations into a row/column FFT and element-by-element multiplications.

**Figure 6:**
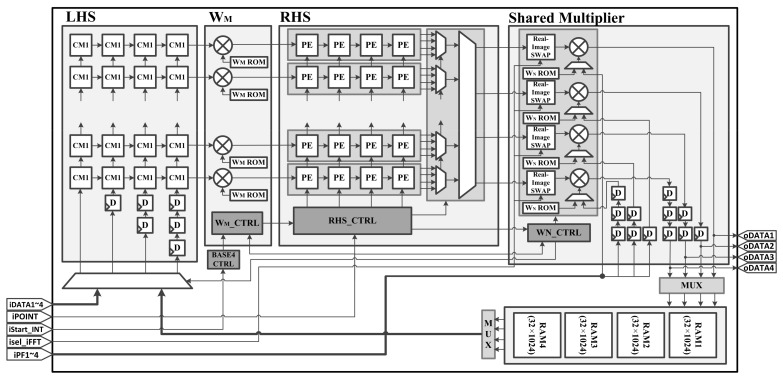
Hardware architecture of the proposed CSA-based SAR processor.

Figure 6 shows the hardware architecture of the proposed CSA-based SAR processor. We adopted a base-4 systolic array that best satisfies the trade-off between area and execution time [27,28]. On the left, there is a bundle of processing element (PE) cells of size $\left(N_{r}/4\right)\times 4$ called left-hand side (LHS), and it is connected to a complex multiplier of size $\left(N_{r}/4\right)\times 1$ that multiplies $W_{M}$. On the right, there is a bundle of PE cells of size $\left(N_{r}/4\right)\times 4$ called right-hand side, (RHS) and it is connected to four shared multipliers that perform $W_{N}$ multiplication or phase compensation operations depending on their input. At the bottom, there are four N/4-sized memories to store the resulting values. Because both $W_{M}$ multiplication and phase compensation operations were element-by-element multiplication, multipliers could be shared. In addition, both operations were performed after the FFT, and the data flow was not disturbed. Therefore, we can achieve area efficiency without using an additional multiplier for the phase compensation operation. Because the proposed hardware supports a maximum of 4096-point operations, the LHS and RHS were PE cells of 16×4 size, and the complex multipliers for $W_{M}$ had a size of 16×1.

The block operation proceeded in the following order: column FFT, $W_{M}$ multiplication, row FFT, and phase compensation. First, the SAR data were transferred to the LHS for column FFT, and matrix multiplication was performed with $C_{M1}$ in the PE cell. By transmitting this result to the $W_{M}$ multiplier, the result of Equation (15) was obtained. Subsequently, the result was transferred to the RHS, and the result of Equation (17) was obtained by performing matrix multiplication with $C_{M2}$ input under the RHS. This result was the same as that for column FFT. The result was transferred to the shared multiplier, and multiplication with the $W_{N}$ was performed. Then, the result was stored in the memory. The data stored in the memory were input to the LHS again in the row direction, and the operation was similarly performed up to the RHS. The result of the RHS was the same as that of FFT and transferred to the shared multiplier. However, unlike before, the phase function was input to the shared multiplier to perform phase compensation. Finally, the result for the FFT and phase compensation operation was stored in the memory.

If the phase factor is 1, it is possible to perform only FFT without phase compensation.

**Figure 7:**
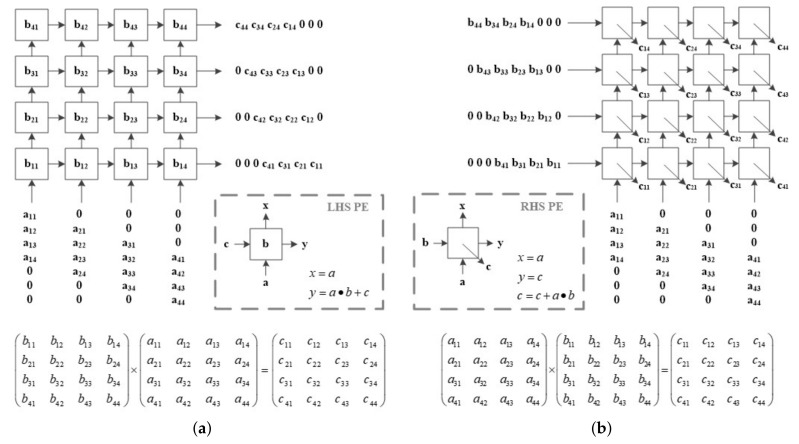
Systolic Array Structure (**a**) LHS (Left Hand Side); (**b**) RHS (Right Hand Side).

In a systolic array, PE cells are locally connected; each PE cell operates simultaneously, and data are delivered to the connected PE cell. It is suitable for algorithms that require a lot of computation because it has a local data flow, and multiple PE cells simultaneously process the computations [25]. A representative operation that can be accelerated using a systolic array is matrix multiplication. Figure 7 shows the two types of PE cells used in the proposed CSA-based SAR processor. For LHS, the data were derived from the lower PE cell, and multiplication and addition operations were performed in each PE cell. It passed through all PE cells by passing the input and the resulting values to each connected PE cell. If matrix A is sequentially input from the bottom, and the B matrix value exists inside the PE cell, B×A can be obtained. For RHS, data were input from the bottom and left cells simultaneously. Similarly, multiplication and addition operations were performed, and the input and the resulting values were transferred to the connected PE cell. After passing through all PE cells, A×B can be obtained. Using the PE array of these structures, the FFT operation expressed by Equations (15) and (17) in a matrix form was performed. Because matrix operations can be performed quickly through systolic arrays, FFT and phase compensation were processed at high speed.

## 4. Implementation and Acceleration Results

**Figure 8:**
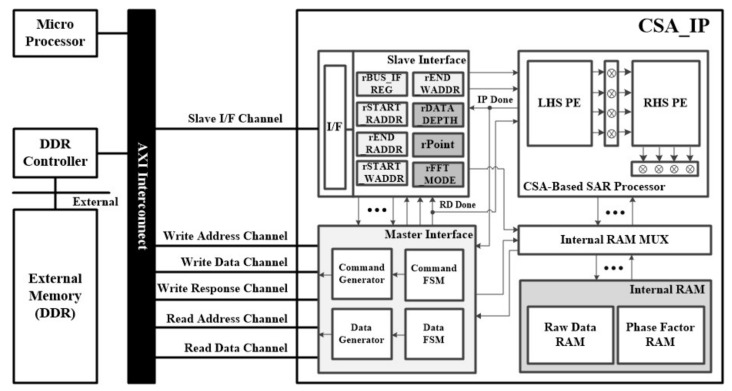
FPGA platform for the verification of the proposed CSA-based SAR processor.

The proposed CSA-based SAR processor was configured on an FPGA platform using an advanced extensible interface (AXI) bus interface for verification. Figure 8 shows the FPGA platform, which includes a CSA-based SAR processor for FFT and phase compensation operations. The system structure comprised a CSA-based SAR processor, master interface to communicate with double data rate (DDR) memory, slave interface to communicate with a microprocessor, and cache RAM to store input/output data and phase functions. In addition, there was a register to change the operation mode because it supported the FFT and IFFT modes and variable lengths from 64 to 4096. The master interface was connected to the DDR memory controller via a 128-bit AXI bus, allowing the transfer of four 32-bit data points per clock cycle. Therefore, it operated efficiently in the base-4 systolic structure, in which four points of data were input in parallel.

**Figure 9:**
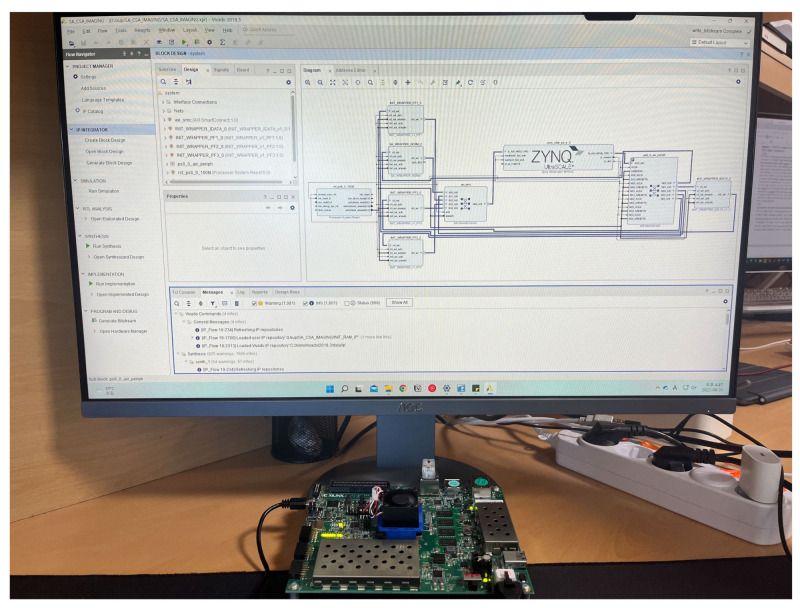
Verification environment for the proposed FPGA implementation.

The proposed CSA-based SAR processor was implemented using a Verilog HDL on a Xilinx Zynq UltraScale+ FPGA device. The CSA-based SAR processor was implemented with 17,326 CLB registers, 31,025 CLB LUTs, 4 block RAMs, and 78 DSPs, as listed in Table 1. The CSA-based SAR processor could process at a maximum operating frequency of 235 MHz, and its power consumption was measured to be 1.31 W. Figure 9 shows the verification environment of the FPGA platform.

When SAR data were loaded into the DDR memory to verify the CSA-based SAR processor, the microprocessor sent a starting signal to the CSA-based SAR processor. The DDR data were then transferred to the cache RAM through the master interface. The CSA-based SAR processor performed azimuth FFT and first-phase compensation operations and stored the result in the cache RAM; the result was transferred back to the DDR via the master interface for the transpose operation. After the transpose operation, the range FFT and second-phase compensation operations were similarly performed. According to the modified CSA flow 2, transposing the result was unnecessary. Therefore, the result was not transmitted to the DDR, and the CSA-based SAR processor performed range IFFT and third-phase compensation operations on the data in the cache RAM and then transmitted the result to the DDR. After performing the transpose operation again, the SAR image was obtained by performing the same operation for the azimuth IFFT. Therefore, SAR images can be obtained by performing four times CSA-based SAR processor operations.

**Figure 10:**
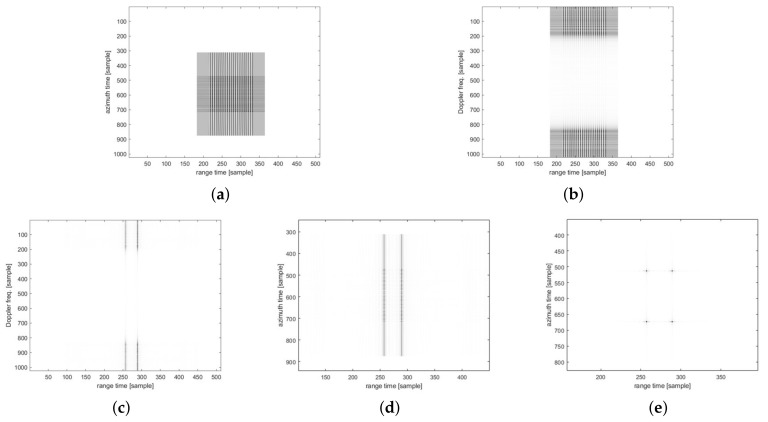
Point target simulation results with traditional CSA flow: (**a**) raw data in time domain; (**b**) differential RCMC result in R/D domain; (**c**) range compression and bulk RCMC result in R/D domain; (**d**) range compression and bulk RCMC result in time domain; (**e**) azimuth compression result in time domain.

**Figure 11:**
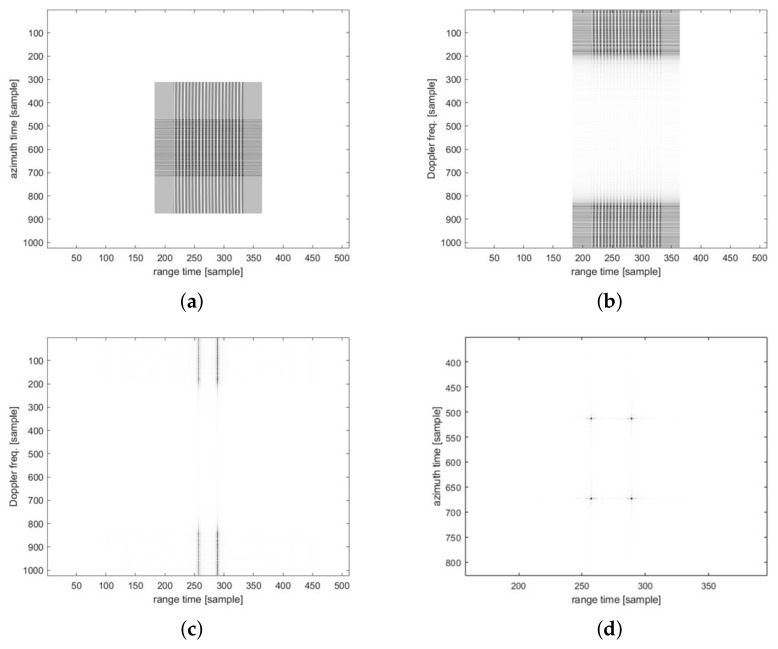
Point target simulation results with modified CSA flow: (**a**) raw data in time domain; (**b**) first block result in R/D domain; (**c**) second block result in R/D domain; (**d**) third block result in time domain.

Figure 10 and Figure 11 show the imaging results for the four-point targets. Figure 10 shows the results of imaging using the traditional CSA flow, and Figure 11 shows the results using the modified CSA flow. The third-phase compensation of the modified CSA flow was performed on the range axis, and the result of completing the range axis operation is shown in Figure 11c. Therefore, as shown in Figure 11d, the imaging result can be obtained through only the azimuth IFFT. However, for traditional CSA flow, a third-phase compensation operation was performed on the azimuth axis. Figure 10c shows the result of completing the range-axis operation, and Figure 10d shows the data of Figure 10c in the time domain. The operation of the range axis was completed, but the azimuth compression had not yet been performed, which was a distinct difference from the modified CSA flow. We analyzed the peak signal-to-noise ratio (PSNR) [32] based on the numerical error and structural similarity index map (SSIM) [33] based on the structural similarity of images as metrics to evaluate the SAR image quality. The PSNR was measured at 35.44 dB, which is higher than 30 dB, and the SSIM was measured at 0.9544.

**Figure 12:**
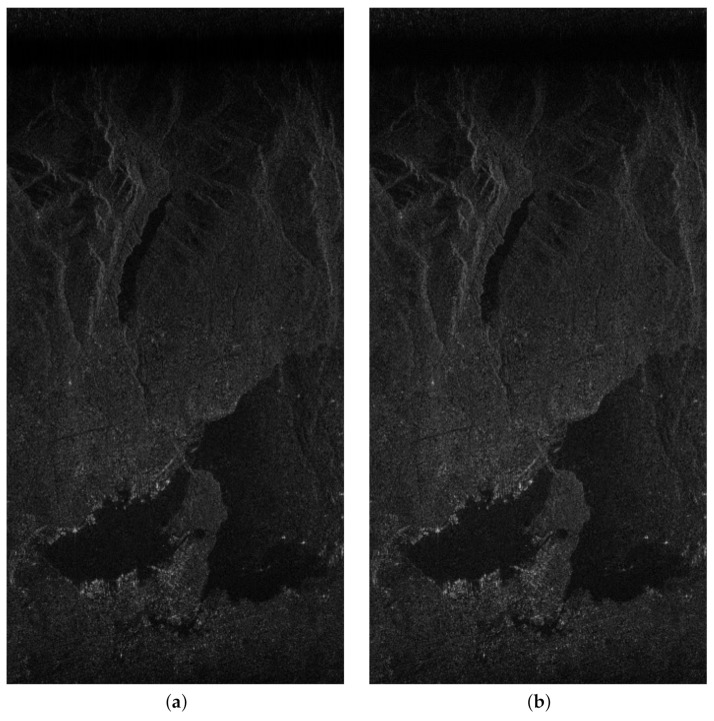
SAR images derived using (**a**) ARM Cortex-A53-based SW and (**b**) the proposed FPGA-based HW.

For validation using actual SAR data, we used the RADARSAR-1 dataset, an image of Vancouver, Canada, from RADARSAT-1’s Fine Beam 2 [31]. The software processing results using ARM Cortex-A53 were used as references to evaluate the image quality of the proposed hardware results. Figure 12 shows the SAR images obtained after processing the actual SAR data. The PSNR and SSIM were measured at 33.43 dB and 0.9466, respectively. Compared with the results for point targets, PSNR and SSIM were slightly degraded because actual SAR data contained clutter and interference. However, the image quality was still good, as shown in Figure 12.

Table 2 presents the evaluation results of the CSA execution time. The acceleration results obtained using the CSA-based SAR processor and ARM Cortex-A53 are presented for various image sizes. According to the modified CSA flow, all CSA operations were accelerated by the CSA-based SAR processor. The experimental results indicate that the execution time decreased from about 267.56 s to 1.96 s for 4096 × 4096-pixel image, resulting in a 136.2-fold acceleration.

Table 3 compares the execution times of the proposed CSA-based SAR processor with previous studies performed on various computing platforms. Because the sizes of the images presented by each study were different, the execution time per pixel is additionally presented for comparison, and the unit is nanoseconds (ns). The authors of [14] achieved the fastest speed using a combination of a CPU and GPU. However, the power consumption was 345 W, which is unsuitable for small platforms. In [16], the authors proposed an array-based heterogeneous processor. Each PE cell performed a four-point butterfly operation, and 512 PE cells were used. Furthermore, additional multipliers were used to perform the phase compensation operation. However, the proposed design did not use other resources for phase compensation operations and used 128 PE cells. Assuming that the 4-point butterfly unit used in [16] used 4 adders and 3 multipliers, 2048 adders and 1536 multipliers were used. In contrast, each PE cell of the proposed design used 1 adder and 1 multiplier; thus, 128 adders and 128 multipliers were used. The difference in the number of calculators used in the PE cell was 16 times for the adder and 12 times the multiplier, which led to a significant difference in execution time (approximately 3.19 times). Therefore, the proposed design could achieve a faster speed per unit area than that in [16]. A comparison of the results is presented in Table 4. Compared with [12,15,17], the proposed architecture achieved a higher speed and consumed less power, making it suitable for small SAR platforms.

## 5. Conclusions

In this study, we proposed a CSA-based SAR processor based on a systolic array. The CSA-based SAR processor supports FFT and phase compensation operations. The multiplier used for the FFT operation was designed to be shared for phase compensation. Therefore, an additional multiplier for phase compensation was not required, and the area efficiency could be achieved. The proposed architecture is suitable for a modified CSA flow, which changes the order of transpose operation from the traditional CSA flow. We confirmed the imaging result using actual SAR data. The proposed processor was implemented using 17,326 CLB registers, 31,025 CLB LUTs, 4 block RAMs, and 78 DSPs on a Xilinx Zynq UltraScale+ FPGA device. Compared with the execution time of the ARM Cortex-A53-based software for an image of 4096 × 4096 pixels, we achieved an approximately 136.2-fold acceleration. We computed the execution time normalized by the number of pixels and compared the results with those of previous studies. Compared with previous studies conducted on various platforms, the CSA-based SAR processor achieved the fastest speed per the number of calculators or power.

Future research will involve the implementation of ASIC usable in small SAR platforms based on the proposed design verified through FPGA. In addition, we expect to implement this model in more power-efficient platforms.

## Figures and Tables

**Table 1:** Implementation results based on the Xilinx Zynq UltraScale+ FPGA device.

Unit	CLB Register	CLB LUT	Block RAM	DSP	Max. Operating Clock Freq.
Systolic Array Unit	17,326	31,025	4	78	235 MHz
LHS	3972	3717	-	0	-
Wb Multiplier	2374	3160	-	62	-
RHS	9952	21,335	-	0	-
Shared Multiplier	950	2287	-	16	-

**Table 2:** CSA execution time.

Image Size	SW (s)	HW (s)	Speedup Ratio
256 × 256	0.74	0.0073	101.37
512 × 512	3.16	0.0297	106.40
1024 × 1024	13.92	0.1191	116.88
2048 × 2048	61.33	0.4796	127.88
4096 × 4096	267.56	1.9645	136.20

**Table 3:** Comparison with previous implementation.

Work	Platform	Operating Freq.	Image Size	Exec. Times (s)	Power	Exec. Time/Pixel (ns)
Proposed	FPGA	235 MHz	4096 × 4096	1.9645	1.31 W	117.09
			2048 × 2048	0.4796		114.35
			1024 × 1024	0.1191		113.58
			512 × 512	0.0297		113.30
			256 × 256	0.0073		111.39
[12]	Microprocessor + FPGA	-	6472 × 3328	8	68 W	371.42
[14]	CPU+GPU	-	32,768 × 32,768	2.8	345 W	2.61
[15]	Mobile-GPU	2.3 GHz	2048 × 2048	3.19	5 W	760.56
[16]	ASIC	200 MHZ	2048 × 2048	0.15	463 mV	35.76
			1024 × 1024	0.04		38.15
[17]	CPU	2.6 GHz	8192 × 8192	13.56	-	202.06

**Table 4:** Comparison of PE unit with [16].

Work	PE Cell Type	The Number of PE Cell	Adders per PE	Multipliers per PE	Total Number of Adders	Total Number of Multipliers
Proposed	Proposed (RHS & LHS)	128	1	1	128	128
[16]	4-Point Butterfly Unit	512	4	3	2048	1536

## Data Availability

Not applicable.
